# Physiology

**Published:** 1878-10

**Authors:** 


### Summary.

*Collaborators:*

Dr. H. Gradle, Dr. L. W. Case, Dr. R. Park,
Dr. R. Tilley, Dr. D. R. Brower.

### Physiology.

Concerning White Corpuscles.—Burdon-Sanderson.—(*Br. Med. Jour.*, Jan. 12, 1878.)

It is now known that fibrin does not exist *as such* in the blood; but is formed by two elements—fibrino-plastic and fibrinogen. The cause of their union has hitherto been obscure, but Burdon-Sanderson has shown that the disintegration of the white corpuscles furnishes a sufficient cause, and that such disintegration takes place as soon as they leave the blood stream. The experimental proof is this: blood is caught in a tall jar which is surrounded by ice; it remains incoagulated, the red corpuscules sink, the white rise and float on the serum. Examination of the upper layer shows the white corpuscles rapidly undergoing disintegration, breaking up into granules which cohere for a time, but eventually disappear. Further, if these leucocytes be withdrawn coagulation is arrested. At a freezing temperature they may be separated by filtration. The filtrate is then absolutely deprived of power of coagulation until the corpuscles be restored to it.

Prof. Schmidt has seen the first formed filaments of fibrin originate from the granular débris left behind by the migrated corpuscules.

Collin (*Le Progrés Médical*), has studied the rationale of the melanæmia and pigmentation of malarial fever. Parts most in contact with the blood (*e. g.* the vascular walls), enjoy the least immunity. These deposits are derived indirectly from the red corpuscles which are destroyed in the spleen. The white corpuscles take up the débris of the red ones and with this form the pigmentation. The immigration in such cases is very active. The process is analagous to transportation of coloring matter by the leucocytes when inflammation is studied by the aid of injection of carmine or similar coloring matter.

Influence of Position on Circulation. — At a June meeting of the Academy of Medicine (*Br. Med. Jour.*, July 13, 1878), Mr. Lister, at the time a visitor in Paris, gave a demonstration of his views on this subject. He held that in the elevated position of a limb, its anæmia was due neither to gravitation of blood nor to paralysis of the vaso-motors, but to reflex action, caused by depletion of the veins and contraction of the arteries. His demonstration consisted of the application of an elastic bandage to the root of the limb, which was kept in an elevated position for a few moments; the arm was then lowered, and although the bandage was retained in place, the arm continued to present a bloodless appearance; finally, the arm was raised again, the bandage removed, while the member was kept for a time in the same position. The redness was seen to rapidly return, and then the limb to get pale again before the application of the tourniquet. Mr. Lister concluded by showing the practical application of the theory, and the good results that may be obtained by the elevation of parts that are the seat of inflammation; he instanced also the good effect of raising the arms above the head in cases of epistaxis, which produces reflex contraction of the vessels of the arms, and sympathetic contraction of those of the head and face.

